# The effect of socioeconomic status on health-care delay and treatment of esophageal cancer

**DOI:** 10.1186/s12967-015-0579-9

**Published:** 2015-07-24

**Authors:** Nana Wang, Fangli Cao, Fang Liu, Yibin Jia, Jianbo Wang, Cihang Bao, Xintong Wang, Qingxu Song, Bingxu Tan, Yufeng Cheng

**Affiliations:** Department of Radiation Oncology, Qilu Hospital of Shandong University, 107 West Wenhua Road, Jinan, 250012 People’s Republic of China; Department of Oncology, Liaocheng People’s Hospital, Liaocheng, People’s Republic of China; Department of Image, Shandong Medical College, Jinan, People’s Republic of China

**Keywords:** Esophageal cancer, Socioeconomic status, Delay, Stage, Treatment

## Abstract

**Background:**

Socioeconomic status (SES) has been focused on as a key determinant of the incidence of cancer, cancer stage at diagnosis as well as treatment choices in western countries. However, to the authors’ knowledge, little work has been done concerning the relationship of SES and esophageal cancer in China.

**Methods:**

Patients diagnosed with primary esophageal cancer from January to December 2007 in Qilu hospital were included. Socioeconomic status was determined by a questionnaire including religion, years of schooling and high education, place of residence, occupation, annual household income, and insurance.

**Results:**

A total of 238 cases were collected in this study. Linear-by-linear association testing revealed that health-care delay was significantly associated with SES (*P* = 0.009). Multivariable logistic regression analysis revealed that increased health-care delay (>2 months) was more frequently observed in patients with lower SES (OR 2.271; 95% CI 1.069–4.853). Patients diagnosed at TNM I and II were more frequently in higher SES groups (*P* = 0.017). The association test was statistically significant for undergoing surgical resection only (*P* = 0.015) and chemotherapy (*P* = 0.015). Multivariable logistic regression analysis revealed that surgical resection only was less performed in higher SES group compared with lower SES group (OR 0.372; 95% CI 0.188–0.734). For chemotherapy, higher SES patients had a three-fold higher likelihood compared with lower SES group (OR 3.042; 95% CI 1.335–6.928).

**Conclusion:**

Socioeconomic status was found to be associated with health-care delay, tumor stage and treatment modalities in esophageal cancer.

**Electronic supplementary material:**

The online version of this article (doi:10.1186/s12967-015-0579-9) contains supplementary material, which is available to authorized users.

## Background

Esophageal cancer is the eighth most common cancer and the sixth leading cause of cancer deaths worldwide. Patients with esophageal cancer have a poor prognosis, and the 5-year overall survival rate is only 15–20% [1]. One of the reasons that lead to this dismal prognosis is diagnosed at a relatively advanced stage of this malignancy, because the beginning symptoms are ignored by patients and delay between the first symptom recognition and the first medical consultation exists [2]. When people realize the discomfort feelings are severe enough to a hospital visit, many patients have missed their best treatment time. For esophageal cancer treatment, surgery is the first option. Besides, radiotherapy and chemotherapy are also available [3]. Treatment regimens are different based on infiltrated depth of primary tumors, lymph node metastasis status, and many other factors.

Socioeconomic status (SES) is found to be closely related with various cancers, such as hepatocellular cancer, breast cancer, colorectal cancer, and so on [4–8]. These studies mainly focus on the association between SES and the incidence of cancer as well as mortality in cancer. Previous study has shown socioeconomic factors have profound influence on delayed reporting and late-stage presentation of late rectal sequelae among Chinese cervical cancer patients [9]. Delayed reporting and late-stage presentation of late rectal sequelae are more prevalent among Chinese cervical cancer patients with low education or poor financial status. It has also been reported that SES is an important factor in treatment choice of cancer. In breast cancer, patients with a higher SES underwent a more appropriate treatment modality compared to patients with a lower SES [10].

In esophageal cancer, some studies have shown a strong association of SES with ESCC in cancer incidence [11–13], staging and treatment decision [14, 15]. However, to our knowledge, little information is available concerning the association between socioeconomic status and delay from symptom recognition to health care. Therefore, in the current study, we aimed to assess the association between SES and delay to health care in esophageal cancer. Meanwhile, we investigated whether SES was correlated with tumor stage and treatment modalities among esophageal cancer patients.

## Methods

### Study population

We searched all of the esophageal cancer patients in the department of thoracic surgery at Qilu hospital from January to December 2007. Patients were included in this study if they had histologic documentation. A total of 248 patients were collected in the present study.

### Data collection

Patients’ characteristics were obtained from hospital electronic recording system, such as gender, tumor location, tumor stage, histological type, and so on. Tumor localization was categorized into four groups by anatomic subsites: lower thoracic, middle thoracic, upper thoracic, or cervical subsite of the esophagus. Tumor stage was based on the clinical TNM classification, according to the sixth and seventh International Union against Cancer editions, as appropriate.

Socioeconomic status of the subjects was defined by religion, years of schooling and high education, place of residence (rural or urban), occupation, annual household income, and insurance. We collected the information concerning the above covariates using a standard questionnaire. After we got the scale of the covariates, SES was determined on a scale of 1–9 including income, dwelling area in meters squared per person, and years of schooling and higher education, tax payments [16]. We regrouped SES into low (1–3), medium (4–6), and high (6–9) categories.

Dates when the patient first experienced the symptoms and the first medical consultation were recorded. Health-care delay was defined as the time interval between the first symptom appearance and first contacting the health-care system. According to the mean value of this time interval, we divided the patients into two groups: those who sought health care within 2 months or less and those who sought medical care more than 2 months after patients first experienced the symptoms that led to diagnosis.

Treatment modalities in this study included resection, postoperative radiotherapy, postoperative chemotherapy, a combination of radiotherapy and chemotherapy after resection. For each subject, it was determined whether esophageal resection, chemotherapy, or radiotherapy had been performed.

### Statistical analyses

Linear-by-linear association testing (Chi square testing) was used to assess associations between SES and health-care delay, tumor staging and treatment modality. Multivariable logistic regression was used to estimate odds ratios (ORs) with 95% confidence intervals (CI) and *P* values. All *P* values were based on two-sided tests of significance. *P* < 0.05 were regarded as statistically significant. Analyses were performed using SPSS statistical software (version 20.0; SPSS Inc, Chicago, IL, USA).

## Results

### Patient characteristics

The current study consisted of 248 patients with a mean age of 60.5 years old. The majority of patients was male (81.9%). Of these patients, 207 (87%) were diagnosed with squamous cell carcinoma (ESCC) while the remaining had a non-squamous cell carcinoma. 27.3% of the patients reported a delay of more than 2 months. The characteristics of the study population and their delayed interval are shown in Additional file 1: Table S1.

### Health-care delay

Additional file 2: Table S2 shows the number of patients with health-care delay per SES group. We found a lower percentage of esophageal cancer patients with more than 2 months delay in higher SES group (*P* = 0.009). In Additional file 3: Table S3, the result of the multivariable logistic regression analysis is shown. With adjustment for age, gender, tumor location, T stage, N stage, and TNM stage, analysis of the association between SES and health-care delay revealed that shorter delay (≤2 months) was significantly associated with a higher SES (OR 2.271; 95% CI 1.069–4.853; *P* = 0.034).

### Tumor stage

Additional file 2: Table S2 presents the number of patients with T, N and TNM stages per SES group. The linear-by-linear association test was not significant for T (*P* = 0.637), N (*P* = 0.788) and TNM stage (*P* = 0.896). However, after we combined TNM I and II groups, The linear-by-linear association test was statistically significant (*P* = 0.017). The results of the multivariable logistic regression analyses are shown in Additional file 4: Table S4. It was found that the adjusted ORs of tumor stage were not statistically significant. The adjustment factors included age, gender, tumor location, and tumor histology.

### Treatment modality

Additional file 2: Table S2 also shows the result of linear-by-linear association between SES and treatment modalities. The association test was statistically significant for undergoing surgical resection only (*P* = 0.015), showing that surgical resection only were less performed in patients with a higher SES. The association test was also statistically significant for undergoing chemotherapy (*P* = 0.015), demonstrating that chemotherapy were more performed in patients with a higher SES. The P values for radiotherapy (*P* = 0.137) and chemoradiotherapy (*P* = 0.107) did not reach the significant level.

Additional file 5: Table S5 shows the results of the multivariable logistic regression analyses. For surgical resection only, the adjusted OR was statistically significant (OR 0.372; 95% CI 0.188–0.734; *P* = 0.004) with confounding factors including age, gender, tumor location, tumor histology, and TNM stage. In other words, the likelihood that a surgical resection only was performed in higher SES group declined by 62.8% compared with lower SES group. For chemotherapy, the adjusted OR was statistically significant (OR 3.042; 95% CI 1.335–6.928; *P* = 0.008), meaning that compared with lower SES group, patients with a higher SES had a three-fold higher likelihood of undergoing chemotherapy. For radiotherapy and chemoradiotherapy, the adjusted ORs were not statistically significant (radiotherapy: OR 1.951; 95% CI 0.972–3.916; *P* = 0.060; chemoradiotherapy: OR 3.098; 95% CI 0.972–9.871; *P* = 0.056).

Additional file 6: Table S6 shows the result of linear-by-linear association between tumor stage and treatment modalities. The association test was statistically significant for undergoing surgical resection only (*P* = 0.007) and chemotherapy (*P* = 0.046). The P values for radiotherapy (*P* = 0.162) and chemoradiotherapy (*P* = 0.155) did not reach the significant level.

## Discussion

In the present study, we determined the role of SES on delay access to health-care system, tumor stage, and treatment choice in esophageal cancer patients. We found that patients with a higher SES had shorter health-care delay, were less likely to be diagnosed at a late stage, and had a higher likelihood of undergoing chemotherapy after surgical resection.

In this study, ESCC accounts for 87.0% and this percentage is line with incidence rate of ESCC in China where the predominant histological type is squamous cell carcinoma. There is strong evidence to support an association between histological type and SES in esophageal cancer. With increasing SES, the incidence of squamous cell carcinoma declines and that of adenocarcinoma increases [12]. The result of our study is not consistent with previous studies. We found no association between the incidence of ESCC and SES in the present study (*P* = 0.642). The incidence difference can be explained that smoking habits and alcohol consumption are more common in lower SES patients, whereas gastroesophageal reflux (GERD) are more prominent in patients with a higher SES. However, in China, healthy habits do not develop with wealth increasing. Smoking, alcohol consumption and stay up all night are common in modern people with different SES. That may be why there is no correlation between SES and squamous cell carcinoma incidence.

Association between SES and delay access to health care has been shown in cervical cancer, lung cancer, breast cancer before [9, 17–19]. Low SES patients undergo a longer time interval between first symptom recognition and first medical consultation. Our result is line with findings in the previous literature. Previous studies about association between SES and esophageal cancer mainly focus on one single factor for SES, such as educational level, annual income, and so on. In this study, we combined the main factors affecting SES and got a SES scale to analyze. So our study adjusted the main SES factors, and the result could reflect SES more exactly.

For the explanation, as reported before, low education is strongly related to poor health literacy and low or limited health literacy is an important risk factor of worse health outcomes [20]. Patients with low education tend to have low awareness of esophageal cancer, therefore they ignore the symptom and is less likely to seek preventive screening measures. Patients with a higher SES, usually with a higher income, seek medical care with less hesitation. Patients with a lower SES have worse financial support and less ability to afford medical care, thus the time delay is prolonged.

Urban–rural divide is still great nowadays in China. High SES group covers more individuals of urban areas. Medical consultation in urban areas is more convenient. They may have more access to medical services than residents of rural areas, which would in turn make them more likely to seek prompt medical care for any health problems [21]. Moreover, health insurances for urban areas offer a higher ratio of reimbursement. These differences may contribute to disparities in health-care delay.

It has previously been shown that patients with a lower SES are diagnosed at a more advanced stage because of unequally access to health care and increased delay between symptom awareness and visiting primary health care in gastrointestinal tumors [22]. The result of correlation between SES and stage in our study is consistent with previous studies. Patients with a higher SES were more likely to be diagnosed at a less advanced stage. It can be explained by the fact that health care is not equally accessible in China, and people with a higher SES seek health care more conveniently. Furthermore, as we mentioned above, higher SES group tend to have a shorter time interval between symptom recognition and visiting primary medical care.

A study conducted in The Netherlands has shown that no statistically significant correlation is found between SES and TNM stage, because health insurance covers almost all people in The Netherlands, resulting in a similar access to health care faculties for all income groups [15]. Apparently this situation was not suitable for China. Meantime this study indicates that access to health care is an important factor for stage difference between SES groups from another perspective.

Differences in treatment modalities have been reported before [14, 15, 23]. It has been shown in esophageal cancer that resection and chemotherapy are more often performed with increasing SES and no correlation is found between SES and radiation therapy [15]. Reasons for this association are summarized in this paper, including differences in attitudes toward invasive procedures, disease severity, access to care [24], differences in undergoing staging procedures [25], and issues related to health insurance [26]. Our results are in agreement with findings from previous studies. We found that patients with a higher SES were less likely to receive surgical resection only and more likely to undergo chemotherapy after surgery. To explain the differences in treatment modalities between different SES classes, educational level must be taken into consideration. With more years of education, patients show better cognition of the malignant disease. Besides, higher SES patients have more advanced health insurance and primary medical care system. Compared with lower SES group, these patients have lighter economic burden. People with higher SES often have official jobs and the employment departments offer regular physical examinations of the staff. So no wonder the signs and symptoms in this group are recognized earlier. And the optimal opportunity for treatment is not missed. Thus the choice of treatment can be better. This might well suggest that patients with a higher SES are more eager to receive chemotherapy to overcome the malignant disease they are suffering from. But in the mind of patients with a lower SES, cancer is such a kind of disease that cannot be improved at all with high treatment cost. Hence they give up before any treatment options. Besides, socioeconomically disadvantaged patients experience barriers to effective treatment, such as inadequate insurance coverage, financial burden, and access to effective therapy [27]. Refusal of radiation therapy may help to explain that no correlation between SES and radiotherapy were found, because decreasing annual income and Asian American race are associated with refusal of radiation [28].

TNM staging system is common used in esophageal cancer and we found significant correlation between TNM staging and treatment modalities. The results showed that surgery only and surgery plus postoperative chemotherapy have significant correlation with tumor staging (*P* =0.007, *P* = 0.046). The reasons under this correlation may be individual index, including different access to medical care, different realization to treatment modalities and issues related to health insurance [24, 26]. Patients with early stage tend to have convenient access to medical care or more aware about their health conditions. As we have discussed before, patients with better SES often have sound health care system, or else they do not have heavy economic burden, so they come to hospital without hesitancy. And stage of tumor is the determining factor for treatment choice, patients diagnosed at an earlier stage can receive prompt therapy with optimal timing.

Our study has several limitations. First, the sample size was small since there were only 238 cases included and only a single institution was investigated. For socioeconomic study, the number was relatively small. Multicenter, large sample clinical analysis is required for further study. Second, the calculation method of SES was self-designed because of the absence of a recognized standard for SES measures in China. Third, the included patients were from the department of thoracic surgery, and all of them underwent operation for treatment. So the differences in treatment modalities mainly focus on additional treatment after surgery. The effect of SES on patients receiving surgery or not cannot be researched from this study.

## Conclusion

We identified an association between SES and delay access to health-care system. Additionally, SES was found to be involved with tumor stage and treatment choice in esophageal cancer. Patients with a higher SES were more likely to undergo chemotherapy after surgery. Further studies are needed to provide more insight in the causes of these differences.

## Additional files

Additional file 1:
**Table S1.** Patient characteristics. Additional file 2:
**Table S2.** Linear-by linear association between SES and health-care delay/tumor stage /treatment. Additional file 3:
**Table S3.** Multivariable logistic regression analysis of between SES and health-care delay. Additional file 4:
**Table S4.** Multivariable logistic regression analysis of between SES and tumor stage. Additional file 5:
**Table S5.** Multivariable logistic regression analysis of between SES and treatment modalities. Additional file 6:
**Table S6.** Linear-by linear association between TNM stage and treatment modalities.
