# Supplementary material for: The effect of socioeconomic status on health-care delay and treatment of esophageal cancer
Source: J Transl Med. 2015 Jul 24;13:241. doi: 10.1186/s12967-015-0579-9 (PMC4511992; doi:10.1186/s12967-015-0579-9)
Supplement: Additional file 1: — Table S1. Patient characteristics. [file 12967_2015_579_MOESM1_ESM.docx]

Table S1 Patient characteristics

|  | characteristics |
| --- | --- |
| Age (mean±SD) | 60.5±8.2 |
| Gender (%)  Male  Female | 195 (81.9)  43 (18.1) |
| Location (%)  Cervical  Upper  Middle  Lower | 9 (3.8)  20 (8.4)  72 (30.3)  137 (57.6) |
| Histology (%)  SCC  Non-SCC  T stage (%)  I  II  III+IV  N stage (%)  No  Yes | 207 (87.0)  31 (13.0)  26 (10.9)  48 (20.2)  164 (68.9)  130 (54.6)  108 (45.4) |
| TNM stage (%)  I  II  III+IV  SES (%)  Low  Medium  High  Delay presentation (%)  ≤2 months  ＞2 months | 31 (13.0)  106 (44.5)  101 (42.4)  96 (40.3)  77 (32.4)  65 (27.3)  173 (72.7)  65 (27.3) |

SCC, squamous-cell carcinoma; SES, socioeconomic status.
