# Supplementary material for: The effect of socioeconomic status on health-care delay and treatment of esophageal cancer
Source: J Transl Med. 2015 Jul 24;13:241. doi: 10.1186/s12967-015-0579-9 (PMC4511992; doi:10.1186/s12967-015-0579-9)
Supplement: Additional file 2: — Table S2. Linear-by linear association between SES and health-care delay/tumor stage /treatment. [file 12967_2015_579_MOESM2_ESM.docx]

Table S2 Linear-by linear association between SES and health-care delay/tumor stage /treatment

|  | Low SES (96) | Medium SES (77) | High SES (65) | *P* value |
| --- | --- | --- | --- | --- |
| Health-care delay  ≤2 months (%)  ＞2 months (%)  Tumor stage  T stage (%)  I  II  III+IV  N stage (%)  No  Yes  TNM stage (%)  I  II  III+IV  TNM stage (%)  I+II  III+IV  Treatment modalities  Surgical resection only (%)  Chemotherapy (%)  Radiotherapy (%)  Chemoradiotherapy (%) | 60 (62.5)  36 (37.5)  9 (9.4)  17 (17.7)  70 (72.9)  52 (54.2)  44 (45.8)  9 (9.4)  43 (44.8)  44 (45.8)  52 (54.2)  44 (45.8)  59 (61.5)  14 (14.6)  29 (30.2)  6 (6.25) | 61 (79.3)  16 (20.7)  10 (13.0)  19 (24.7)  48 (62.3)  48 (62.3)  29 (37.7)  15 (19.5)  39 (50.6)  23 (29.9)  54 (70.1)  23 (29.9)  52 (67.5)  14 (18.2)  17 (22.1)  6 (7.79) | 52 (80.0)  13 (20.0)  7 (10.8)  12 (18.5)  46 (70.8)  33 (50.8)  32 (49.2)  7 (10.8)  24 (36.9)  34 (52.3)  31 (47.7)  34 (52.3)  26 (40.0)  20 (30.8)  28 (43.1)  9 (13.8) | 0.009  0.637  0.788  0.896  0.017  0.015  0.015  0.137  0.107 |

SES: socioeconomic status
