# Supplementary material for: The effect of socioeconomic status on health-care delay and treatment of esophageal cancer
Source: J Transl Med. 2015 Jul 24;13:241. doi: 10.1186/s12967-015-0579-9 (PMC4511992; doi:10.1186/s12967-015-0579-9)
Supplement: Additional file 3: — Table S3. Multivariable logistic regression analysis of between SES and health-care delay. [file 12967_2015_579_MOESM3_ESM.docx]

Table S3 Multivariable logistic regression analysis of between SES and health-care delay

|  | *P* value | OR | 95% CI |
| --- | --- | --- | --- |
| Health-care delay | 0.034 | 2.271 | 1.069-4.853 |

Covariates: Age, gender, tumor location, T stage, N stage, TNM stage and SES.

OR, odds ratio; SES, socioeconomic status.
