# Supplementary material for: The effect of socioeconomic status on health-care delay and treatment of esophageal cancer
Source: J Transl Med. 2015 Jul 24;13:241. doi: 10.1186/s12967-015-0579-9 (PMC4511992; doi:10.1186/s12967-015-0579-9)
Supplement: Additional file 4: — Table S4. Multivariable logistic regression analysis of between SES and tumor stage. [file 12967_2015_579_MOESM4_ESM.docx]

Table S4 Multivariable logistic regression analysis of between SES and tumor stage

| Tumor stage | *P* value | OR | 95% CI |
| --- | --- | --- | --- |
| T stage  I  II  III+IV  N stage  No  Yes  TNM stage  I  II  III+IV  TNM stage  I+II  III+IV | 0.904  0.794  -  0.255  -  0.651  0.780  -  0.639  - | 1.069  1.121  1.000  0.684  1.000  0.770  0.904  1.000  1.170  1.000 | 0.361-3.166  0.474-2.651  Reference  0.355-1.316  Reference  0.249-2.384  0.446-1.834  Reference  0.607-2.254  Reference |

Covariates: Age, gender, tumor location, tumor histology and SES.

OR, odds ratio; SES, socioeconomic status.
