# Supplementary material for: The effect of socioeconomic status on health-care delay and treatment of esophageal cancer
Source: J Transl Med. 2015 Jul 24;13:241. doi: 10.1186/s12967-015-0579-9 (PMC4511992; doi:10.1186/s12967-015-0579-9)
Supplement: Additional file 5: — Table S5. Multivariable logistic regression analysis of between SES and treatment modalities. [file 12967_2015_579_MOESM5_ESM.docx]

Table S5 Multivariable logistic regression analysis of between SES and treatment modalities

| Treatment modalities | *P* value | OR | 95% CI |
| --- | --- | --- | --- |
| Surgical resection only  Chemotherapy  Radiotherapy  Chemoradiotherapy | 0.004  0.008  0.060  0.056 | 0.372  3.042  1.951  3.098 | 0.188-0.734  1.335-6.928  0.972-3.916  0.972-9.871 |

Covariates: Age, gender, tumor location, tumor histology, TNM stage and SES.

OR, odds ratio; SES, socioeconomic status.
