# Supplementary material for: The effect of socioeconomic status on health-care delay and treatment of esophageal cancer
Source: J Transl Med. 2015 Jul 24;13:241. doi: 10.1186/s12967-015-0579-9 (PMC4511992; doi:10.1186/s12967-015-0579-9)
Supplement: Additional file 6: — Table S6. Linear-by linear association between TNM stage and treatment modalities. [file 12967_2015_579_MOESM6_ESM.docx]

Table S6 Linear-by linear association between TNM stage and treatment modalities

| Treatment modalities | I (31) | II (106) | III+IV (101) | *P* value |
| --- | --- | --- | --- | --- |
| Surgical resection only (%)  Chemotherapy (%)  Radiotherapy (%)  Chemoradiotherapy (%) | 20 (64.5)  8 (25.8)  6 (19.4)  3 (9.68) | 68 (64.3)  19 (17.9)  29 (27.4)  10 (9.43) | 49 (48.5)  21 (19.8)  39 (38.6)  8 (7.92) | 0.007  0.046  0.062  0.155 |
